# Soluble Co-Signaling Molecules Predict Long-Term Graft Outcome in Kidney-Transplanted Patients

**DOI:** 10.1371/journal.pone.0113396

**Published:** 2014-12-05

**Authors:** Susana G. Melendreras, Pablo Martínez-Camblor, Aurora Menéndez, Cristina Bravo-Mendoza, Ana González-Vidal, Eliecer Coto, Carmen Díaz-Corte, Marta Ruiz-Ortega, Carlos López-Larrea, Beatriz Suárez-Álvarez

**Affiliations:** 1 Department of Nephrology, Hospital Universitario Central de Asturias, Oviedo, Spain; 2 Department of Nephrology, Hospital Son Llátzer, Palma de Mallorca, Spain; 3 Oficina de Investigación Biosanitaria de Asturias (OIB-FICYT), Oviedo, Spain; 4 Universidad Autónoma de Chile, Santiago de Chile, Chile; 5 Department of Immunology, Hospital Universitario Central de Asturias, Oviedo, Spain; 6 Genética Molecular-Laboratorio de Medicina, Hospital Universitario Central de Asturias, Oviedo, Spain; 7 Cellular Biology of Renal Diseases Laboratory, Instituto de Investigación Sanitaria Fundación Jiménez Díaz, Universidad Autónoma Madrid, Madrid, Spain; 8 Fundación Renal Iñigo Álvarez de Toledo, Madrid, Spain; San Raffaele Hospital, Italy

## Abstract

Co-signaling molecules are responsible for full T-cell activation after solid organ transplantation. Their increased expression can lead to the release of a soluble form that can modulate the immune response post-transplantation. We analyzed the presence of co-signaling molecules (sCD30, sCD40, sCD137, sCTLA-4, sCD80, sCD28, sCD40L, sPD-1, and sPD-L1) in serum from kidney-transplanted patients (n = 59) obtained at different times (before transplantation, and 15 days, 3 months and 1 year post-transplantation) and their contribution to graft outcome was evaluated using principal component analysis. Before transplantation, high levels of soluble co-signaling molecules (mainly sCD30, sCD137 and sCD40) were detected in all patients. These molecules were modulated soon after receiving an allograft but never attained similar levels to those of healthy controls. A signature based on the determination of six soluble co-stimulatory (sCD30, sCD40, sCD137 and sCD40L) and co-inhibitory (sPD-1 and sPD-L1) molecules at 3 months post-transplantation allowed a group of patients to be identified (27.12%) with a worse long-term graft outcome. Patients with high levels of soluble molecules showed a progressive and gradual deterioration of kidney function (increased creatinine and proteinuria levels and decreased estimated glomerular filtration rate) over time and a higher risk of graft loss at 6 years post-transplantation than patients with low levels of these molecules (62.55% *versus* 5.14%, p<0.001). Thus, our data show an aberrant expression of soluble co-signaling molecules in kidney-transplanted patients whose quantification at 3 months post-transplantation might be a useful biomarker of immune status and help to predict long-term graft evolution.

## Introduction

Activation and differentiation of naive CD4 and CD8 T cells are key processes in the development of the immune response to an allograft. After engagement of the T-cell receptor (TCR) with its antigen on-antigen-presenting cells (APCs), a second signal is necessary for full T-cell activation. This signal is sent by the co-stimulatory molecules, which are responsible for clonal T-cell expansion and differentiation, and finally for triggering effector functions. Following initial activation, co-inhibitory molecules are induced to counteract and avoid an exacerbated activation state. In this way, the set of co-stimulatory and co-inhibitory signals and their expression in time and space (cell type) determine the strength, nature and duration of the immune response during transplantation [1]–[3]. Most co-signaling molecules are structurally classified into one of two major families: a) the immunoglobulin superfamily (B7/CD28 family) (e.g., CD28, CTLA-4, ICOS, PD-1), which is involved in triggering the cell-mediated immune response, or b) the tumor necrosis factor receptor (TNFR) family (e.g., CD40, 4-IBB, OX40, CD27, GITR, CD30), whose members are induced later and are involved in the later phases of T-cell activation [4]–[6]. A third family of cell surface type I transmembrane glycoproteins (e.g., TIM1, TIM3, TIM4) also have important immunoregulatory functions [7]–[9].

Co-stimulation blockade is thought to selectively modulate the immune response after transplantation [10]–[15]. One of the most promising therapeutic approaches has been the use of belatacept, a human CTLA4-Ig fusion protein recently approved by the FDA as a primary immunosuppressant in kidney transplantation [16], [17]. Other co-signaling blockers are currently in the under investigation or evaluation in clinical trials [18]. Blockage of the CD160-CD160L interaction using a CD160 fusion protein (CD160Ig), in combination with rapamycin and CTLA4-Ig, has been successful to prolong cardiac allograft survival [19]. The future success of co-signaling blockage in clinical transplantation will depend on the combined use with other therapeutic agents and the need to be modified during the specific stages of the immune response.

Aberrant expression of soluble co-stimulatory molecules is associated with persistent activation of T cells in autoimmune diseases [20]–[23]. However, the role of these soluble molecules during transplantation is not fully understood. In this study, we examined whether the presence of soluble co-signaling molecules might reveal the immunological status of kidney-transplanted patients and help predict graft outcome. We observed that patients with high levels of co-stimulatory (sCD30, sCD40, sCD137, sCD40L) and/or co-inhibitory (sPD-1, and sPD-L1) molecules at 3 months post-transplantation were associated with a significantly higher risk of graft failure at 6 years post-transplantation. Thus, our results show that the levels of these soluble molecules might be a useful biomarker for predicting long-term graft function.

## Material and Methods

### Patients and Samples

Sera from 59 deceased donor kidney recipients transplanted consecutively during 2006 and 2007 in the Hospital Universitario Central de Asturias, Spain, were collected at different times (before transplantation, and 15 days, 3 months and 1 year after transplantation). Sera were obtained on all occasions for all patients enrolled in the study. All patients gave their written informed consent. The study adhered to the Principles of the Declaration of Helsinki and Istanbul and the obtained approval from the local ethics committee (Comite Ético de Investigación Clínica Regional del Principado de Asturias). Serum from 25 adult healthy blood donors (mean age 49.8±15.3 years; male∶female 9∶16) was obtained from the Blood Transfusion Center, Oviedo, Spain, after informed consent. All sera samples were stored at −80°C until analysis and repeated freezing/thawing cycles were avoided. Thirty-seven (62.7%) of the patients received induction therapy, 55.93% with thymoglobulin and 6.77% with basiliximab (anti-IL2R). All patients received therapy with steroids and calcineurin inhibitors (44.06% cyclosporine and 55.93% tacrolimus), 84.74% mycophenolate mofetil (MMF) and 6.77% mammalian target of rapamycin (mTOR) inhibitors. A total of 91.53% of the patients were on a triple therapy immunosuppression regimen (84.74% MMF and 6.77% mTOR inhibitors) and only five of them (8.47%) were on a double therapy regimen. Further demographics and clinical characteristics of all transplanted patients are summarized in **Table 1**. The estimated glomerular filtration rate (eGFR) was calculated with the Modification of Diet in Renal Disease (MDRD-IDMS) formulae: eGFR (mL/min/1.73 m^2^)  = 175 x (creatinine)^−1.154^ x (age)^−0.203^ x (0.742 if female) x (1.2010 if black) [24]. HLA typing of donors and recipients and a complete screening for HLA antibodies were carried out for all patients using Lifecodes HLA-SSO typing kits (Tepnel Lifecodes Corporation, Stamford, UK) and LABscreen kit (One Lambda, Inc., Canoga Park, CA) based on Luminex xMAP technology (Luminex Corp, Austin, TX), following the manufacturer's instructions. Graft function was documented at each sampling time and annually thereafter until the sixth year post-transplantation. Graft loss was defined as the absence of kidney function due to irreversible graft injury and return of the patients to dialysis.

**Table 1:** Demographic characteristics of kidney-transplanted patients.

Characteristics	Patients (n = 59)
Donor age (years)	51±17.5 (13–80)
Donor gender, Female, n (%)	29 (49.15)
Recipient age (years)	51.8±14.3 (16–77)
Recipient gender, Female, n (%)	17 (28.81)
Time on dialysis (months)	19.3±15.3
Type of dialysis, n (%):	
Hemodialysis (HD)	41 (69.49)
Peritoneal dialysis (PD)	15 (25.42)
Predialysis	3 (5.08)
Cause of ESRD, n (%):	
Vascular nephropathy	7 (11.86)
Diabetes mellitus	10 (16.94)
Polycystic kidney disease	10 (16.94)
Glomerulonephritis	15 (25.42)
Pyelonephritis/interstitial nephritis	8 (13.55)
Unknown	4 (6.77)
Other	5 (8.47)
Previous transplant, n (%)	8 (13.55)
Number of HLA mismatches, n (%):	
0–2	5 (8.47)
3–4	36 (61.01)
5–6	18 (30.50)
Pre-transplantation DSA/no DSA, n (%)	0/5 (8.47)
Acute rejection, n (%)	6 (10.16)
Induction therapy, n (%)	
None	22 (37.28)
Basiliximab	33 (55.93)
Thymoglobulin	4 (6.77)
Immunosuppressive therapy, n (%)	
Steroids	59 (100)
Cyclosporine	26 (44.06)
Tacrolimus	33 (55.93)
Mycophenolate mofetil	50 (84.74)
mTOR inhibitors	4 (6.77)

Continuous variables are summarized as mean ± standard deviation (interquartile range). Categorical variables are shown as n (%). ESRD, end-stage renal disease; mTOR, mammalian target of rapamycin, DSA, donor-specific antibody.

### Detection of Soluble Co-signaling Molecules by ELISA

Soluble co-signaling molecules were measured in serum samples by the ELISA technique. ELISA kits for soluble CD28 (BMS290), CD30 (BMS240MST), CD40 (BMS265MST), CD40L (BMS239MST), CD80 (BMS291INST), CD137 (BMS289), CTLA-4 (BMS276MST), and FAS (BMS245MST) were obtained from Bender MedSystems, Vienna, Austria, and used according to the manufacturer's instructions.

For soluble PD-1 and PD-L1 (B7-H1), microtiter plates were coated overnight at 4°C with 1 µg/ml of anti-human PD-1 antibody (AF1086, R&D Systems, UK) or anti-human B7-H1 antibody (AF156, R&D Systems). After blocking, serum samples were diluted 1∶4 for sPD-1 and 1∶2 for sPD-L1 molecule detection and purified recombinant human PD-17Fc (1086-PD, R&D Systems) and B7-H17Fc (156-B7, R&D Systems) chimeras were used as standards. Further, plates were incubated with 1 µg/ml of anti-human PD-1 monoclonal antibody (mAb, MAB1086, R&D Systems) or B7-H1 mAb (MAB156, R&D Systems), washed and a solution of conjugated goat anti-mouse IgG was added. Color reactions were developed using the TMB peroxidase substrate system (Sigma-Aldrich, St. Louis, MO) and measured at 450 nm. All samples were analyzed in duplicate and standard curves were run in each plate. No cross-reactivity between PD-1 and PD-L1 molecules was observed and intra- and inter-assay variability was evaluated to be <9% in all cases.

### Statistical Analysis

Demographic and clinical data were summarized as the mean and standard deviation (SD) or a percentage, and non-normally distributed soluble molecular data were described by their median and interquartile range (IQR). Unpaired and paired Wilcoxon, Mann–Whitney U and chi-squared tests or Spearman correlation were used to compare variables. Data from soluble co-signaling molecules were reduced by principal component analysis (PCA) of the correlation matrix; missing values were replaced by the median of the variable. The scoring variables obtained (PC1 and PC2) were used in a cluster analysis to identify distinct patient groups. Due to the existence of competing events, we studied the incidences of graft loss using the Nelson-Aalen estimator. We developed a standard Cox regression model to estimate cause-specific HRs. Data were analyzed with R.2.15 (www.r-project.org). Differences were considered to be significant for values of p<0.05.

## Results

### Soluble Co-signaling Molecule Kinetics during Kidney Transplantation

**Figure 1:**
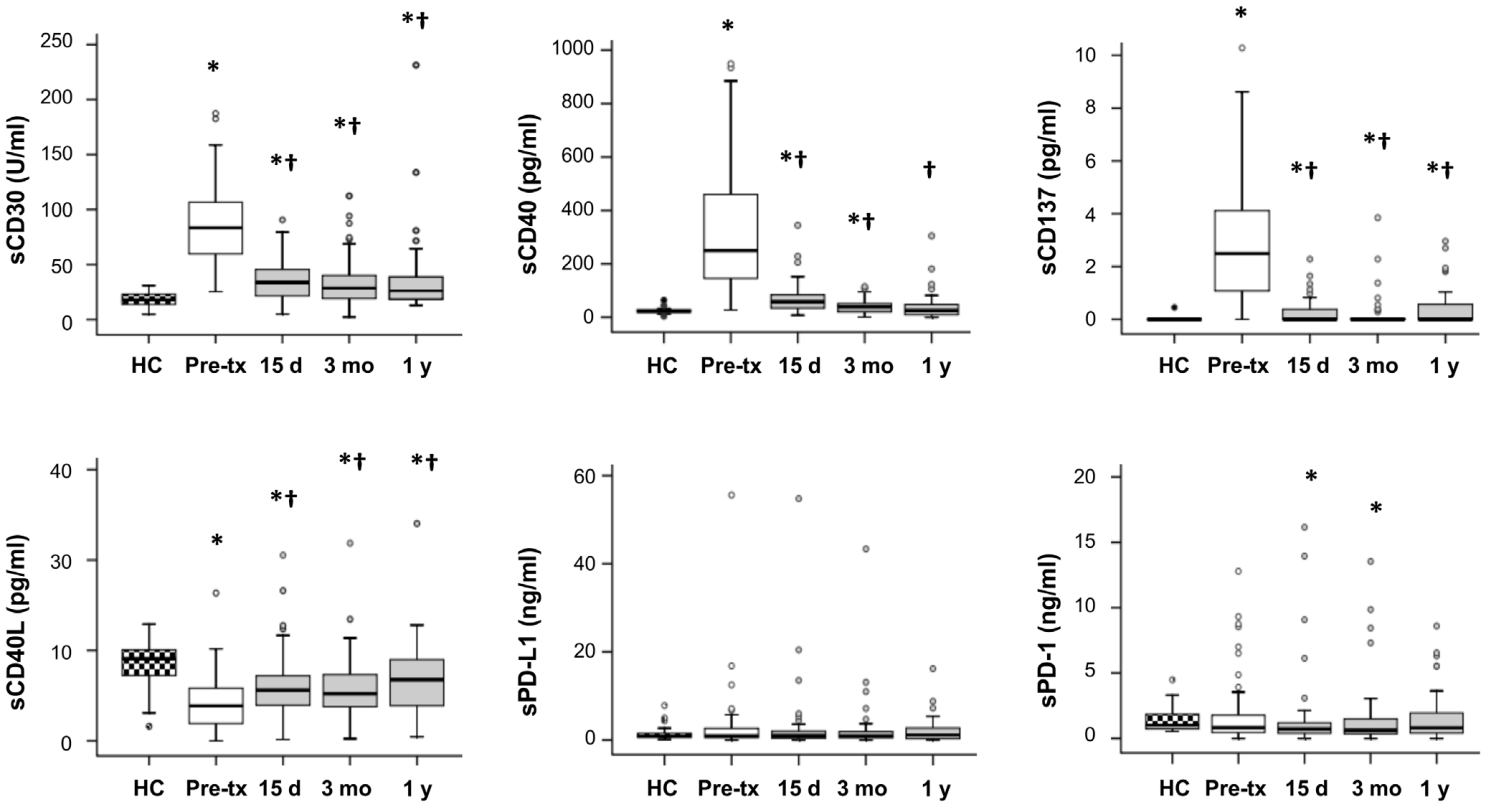
Modulation of soluble co-signaling molecules in kidney-transplanted patients over time. The levels of the soluble co-signaling molecules CD30, CD40, CD137, CD40L, PD-1 and PD-L1 were assayed by ELISA in serum samples of healthy controls (n = 25) and kidney-transplanted patients (n = 59) obtained at different times: just before transplantation, and 15 days, 3 months and 1 year after transplantation. Data are shown as box-plots, in which the horizontal line within each box represents the median, the bottom and top of each box represent the 25^th^ and 75^th^ percentiles, the bars represent the 10^th^ and 90^th^ percentiles and circles indicate outliers. Unpaired and paired Wilcoxon tests were used to compare distributions between independent and dependent groups, respectively. * indicates statistically significant differences between healthy controls and kidney-transplanted patient samples, and † indicates statistically significant differences between patients samples obtained at different pre- and post-transplantation times.

We analyzed the presence of several soluble co-signaling molecules in serum from healthy controls (n = 25) and kidney-transplanted patients (n = 59) obtained just before transplantation surgery and at different post-transplantation times. Before receiving a kidney transplant, all patients showed aberrant levels of the T-cell activation-associated sCD30, sCD40, and sCD137 molecules (**Fig. 1** **and** **Table S1**). Low levels of sCD30, sCD40 and sCD137 were found in the serum of healthy controls, whilst we observed 4.5-, 14.8- and 142-fold increases, respectively, in pre-transplantation sera. By contrast, the level of sCD40L was higher in the control group (8.41±2.67 ng/ml) than in pre-transplantation samples (4.26±3.01 ng/ml). No significant differences between the two groups were found for other molecules such as sCTLA-4, sCD80 and sCD28 (data not shown). The soluble PD-1 inhibitory molecule and its ligand PD-L1 were detected at very low levels in all analyzed samples, both healthy controls and patients, although some of the latter showed an unusually high expression of these molecules even before transplantation.

No significant association was observed between the levels of these soluble molecules (individually analyzed at different cut-offs) assayed in the pre-transplantation period and the presence of a previous transplant, dialysis time and type or end-stage renal disease (ESRD) etiology. This result suggests that patients receiving kidney replacement therapy (dialysis) who are awaiting a kidney transplant showed an abnormal presence of soluble co-signaling molecules that could alter the immune response after transplantation.

Furthermore, the levels of soluble co-signaling molecules were measured at different post-transplantation times (15 days, 3 months and 1 year) in the same patients in order to analyze their modulation over time. We observed that the presence of soluble CD30, CD40, and CD137 molecules was even significantly decreased at 15 days post-transplantation in all patients although the levels were always significantly higher than those in the sera of healthy donors (**Fig. 1**). Quantification of levels of these molecules at later times (3 months and 1 year post-transplantation) showed only slight variations, but some patients maintained elevated levels of these molecules during the first year post-transplantation. In contrast, the sCD40L levels were increased immediately after transplantation, being significantly higher at 15 days than pre-transplantation levels (6.19±3.50 ng/ml *versus* 4.26±3.01 ng/ml, p = 0.001). Good correlations for sPD-1 and sPD-L1 were observed between the pre- and post-transplantation times (data not shown), showing that patients with high levels of these co-inhibitory molecules in the pre-transplantation period maintained these levels even after receiving a kidney.

In conclusion, high levels of soluble co-signaling molecules detected before transplantation were modulated soon after receiving a kidney graft but did not reach similar levels to those of healthy controls, and remained aberrantly elevated in some patients.

### Clustering of Soluble Co-signaling Molecules Identifies Two Groups of Kidney-Transplanted Patients

To determine whether the altered levels of these soluble molecules in the post-transplantation period could be useful for identifying patients with different graft outcomes, we analyzed the association between the level of each molecule and several clinical parameters (HLA mismatches, creatinine and proteinuria levels, estimated glomerular filtration rate, acute rejection, graft survival and immunosuppressive therapy). No significant associations were found when co-signaling molecules were analyzed independently with respect to these variables. The sex and age of donors and recipients had no discernible effect on the presence of these soluble molecules.

**Figure 2:**
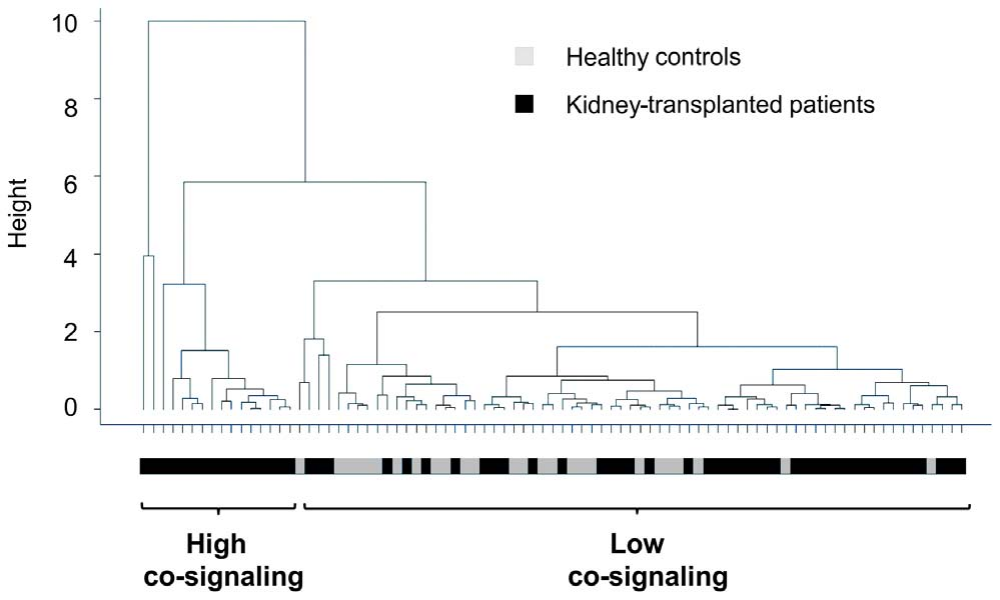
Clustering of patients by levels of co-signaling molecules. Unsupervised two-dimensional hierarchical clustering was used to group patients and healthy controls based on the principal components analysis (PCA) at 3 months post-transplantation. This analysis reduced the soluble molecule data to two principal components (PCs): the co-stimulatory (sCD30, sCD40, sCD137 and sCD40L) and the co-inhibitory (sPD-1 and sPD-L1). The dendrogram is derived from hierarchical clustering of all patients (n = 59) and healthy controls (n = 25) based on the PCs. Each line represents a single kidney-transplanted patient (black) or healthy control (gray). At 3 months post-transplantation, patients overlapping with healthy controls are characterized by low levels of co-stimulatory and co-inhibitory components (low co-signaling group), whilst patients grouped separately from controls are characterized by high levels of co-stimulatory and/or co-inhibitory components (high co-signaling group).

We analyzed the combined effect of assayed soluble molecules using principal component analysis (PCA), which reduces the dimensionality of data and identifies underlying variables that most efficiently explain the variation in the data [25], [26]. In our study, we reduced the information from six soluble molecules to two principal components (PCs) corresponding to their functional characteristics: PC1, the co-stimulatory component, included the sCD30, sCD40, sCD137 and sCD40L molecules; PC2, the co-inhibitory component, included the sPD-1 and sPD-L1 molecules. To check the reliability of this analysis it was necessary to examine whether kidney-transplanted patients were grouped into different categories based on the established PCs. When unsupervised two-dimensional hierarchical clustering was done based on the pre-transplantation levels of the soluble molecules, we were able to show that all patients were grouped separately from healthy controls; some patients were located near healthy controls, whereas others were more distant (**Fig. S1A**). Clustering of the patients based on the levels of co-signaling molecules detected 15 days post-transplantation indicated that some transplanted patients began to overlap with healthy controls (**Fig. S1B**). This became even more evident when the presence of soluble molecules was quantified 3 months post-transplantation (**Fig. 2**), whereby most of the patients were mixed with the healthy controls, although a small group had PC1 and PC2 values that clustered them separately from the controls. At this time, patients mixed with the healthy controls were characterized by low levels of co-stimulatory and co-inhibitory components (low co-signaling group), whilst patients grouped separately from controls were classified by high levels of co-stimulatory and/or co-inhibitory molecules (high co-signaling group). At 1 year post-transplantation, the combined analysis of these molecules was not able to classify patients into different groups, suggesting that attempts to quantify these molecules at later times are not worthwhile.

The components at 3 months post-transplantation were defined by the equations: co-stimulatory component (PC1)  = 0.023 sCD30+0.169 sCD137+0.021 sCD40 - 0.141 sCD40L; co-inhibitory component (PC2)  = 0.266 sPD-1 +0.111 sPD-L1. Patients in the low co-signaling group (n = 43, 72.88%) were classified by co-stimulatory and co-inhibitory component values ≤1.5 whilst patients in the high co-signaling group (n = 16, 27.12%) are defined by co-stimulatory and/or co-inhibitory component values >1.5.

These two groups of patients had characteristics independent of age and sex of donor and recipient, previous transplants, ESRD etiology, HLA mismatches, post-transplantation DSA, delayed graft function and acute rejection (**Table 2**). However, we observed that patients undergoing hemodialysis were significantly more common in the high co-signaling group (68.75% *versu*s 30.23%, p = 0.017) whilst patients in peritoneal dialysis were mainly present in the low co-signaling cluster, although this distinction was not statistically significant. In our study cohort, only three patients were transplanted without renal replacement therapy and although all they showed low levels of co-signaling molecules 3 months after transplantation, it is difficult to obtain reliable conclusions. Surprisingly, we did not find any differences between the two groups based on the received induction therapy. However, patients in maintenance therapy with cyclosporine (CsA) had higher levels of co-signaling molecules at 3 months (high co-signaling group) than those who were receiving tacrolimus (p = 0.037), based on a triple therapy immunosuppressive regimen and independent of the antiproliferative agent (MMF or mTOR inhibitors).

**Table 2:** Clinical and demographic characteristics of patients grouped by levels of soluble co-signaling molecules at 3 months post-transplantation.

	High co-signaling n = 16 (%)	Low co-signaling n = 43 (%)	p
Donor age ≥60 y	8 (50.00)	13 (30.23)	0.222
Female donor	8 (50.00)	21(48.84)	1.000
Recipient age ≥60 y	9 (56.25)	14 (32.55)	0.135
Female recipient	3(18.75)	14 (32.55)	0.352
Previous transplant	3 (18.75)	5 (11.63)	0.670
Type of dialysis:			
Hemodialysis (HD)	11 (68.75)	13 (30.23)	0.017
Peritoneal dialysis (PD)	5 (31.25)	27 (62.79)	0.062
Predialysis	0	3 (6.97)	0.676
ESRD etiology:			0.670
Vascular nephropathy	2 (12.50)	5 (11.62)	
Diabetes mellitus	3 (18.75)	7 (16.27)	
Polycystic kidney disease	0	10 (23.26)	
Glomerulonephritis	5 (31.25)	10 (23.26)	
Pyelonephritis	3 (18.75)	5 (11.62)	
Unknown	1 (6.25)	3 (6.97)	
Others	2 (12.50)	3 (6.97)	
Delayed graft function	7 (43.75)	12 (27.90)	0.347
HLA mismatches ≥5–6	4 (25.00)	13 (30.23)	0.758
Post-transplantation DSA/no DSA at 3 mo	0/2 (12.50)	0/4 (9.30)	1.000
Acute rejection episode in first 3 mo	2 (12.50)	4 (9.30)	1.000
Induction therapy			
None	4 (25.00)	18 (41.86)	0.364
Basiliximab	12 (75.00)	21 (48.84)	0.085
Thymoglobulin	0	4 (9.30)	0.328
Immunosuppressive therapy			
CsA	11 (68.75)	15 (34.88)	0.037
FK506	5 (31.25)	28 (65.12)	0.037
MMF	13 (81.25)	37 (86.05)	0.692
mTOR inhibitors	3 (18.75)	1 (2.33)	0.056
eGFR (ml/min/1.73 m^2^) at 3 mo	51.47±24.71	51.17±17.21	0.682
Serum Cr (mg/dL) at 3 mo	1.54±0.50	1.47±0.43	0.656
Total Pr (g/24 h) at 3 mo	0.46±0.56	0.23±0.17	0.092

Continuous variables are summarized as mean ± standard deviation. Categorical variables are summarized as n (%). CsA, cyclosporine; MMF, mycophenolate mofetil; FK506, tacrolimus; eGFR, estimated glomerular filtration rate, Cr, creatinine; Pr, protein in 24 h urine collection.

In order to analyze whether high levels of soluble co-signaling molecules might be a consequence of impaired graft function, we determined the serum creatinine and proteinuria and estimated glomerular filtration rate (eGFR) at the same time that these molecules were assayed. No significant differences were observed between the two groups for creatinine (1.54±0.50 *versus* 1.47±0.43 mg/dL, p = 0.656) and eGFR (51.47±24.71 *versus* 51.17±17.21 mL/min/1.73 m^2^, p = 0.682), suggesting that quantification of the soluble co-signaling molecules at the 3 months post-transplantation come together patients independently of the kidney function. Patients in the high co-signaling group showed slightly higher levels of proteinuria than the low co-signaling group (0.46±0.56 *versus* 0.23±0.17 g/24 h, p = 0.092), although the difference was not statistically significant.

### Soluble Co-signaling Molecules Predict the Evolution of Kidney Transplantation

**Figure 3:**
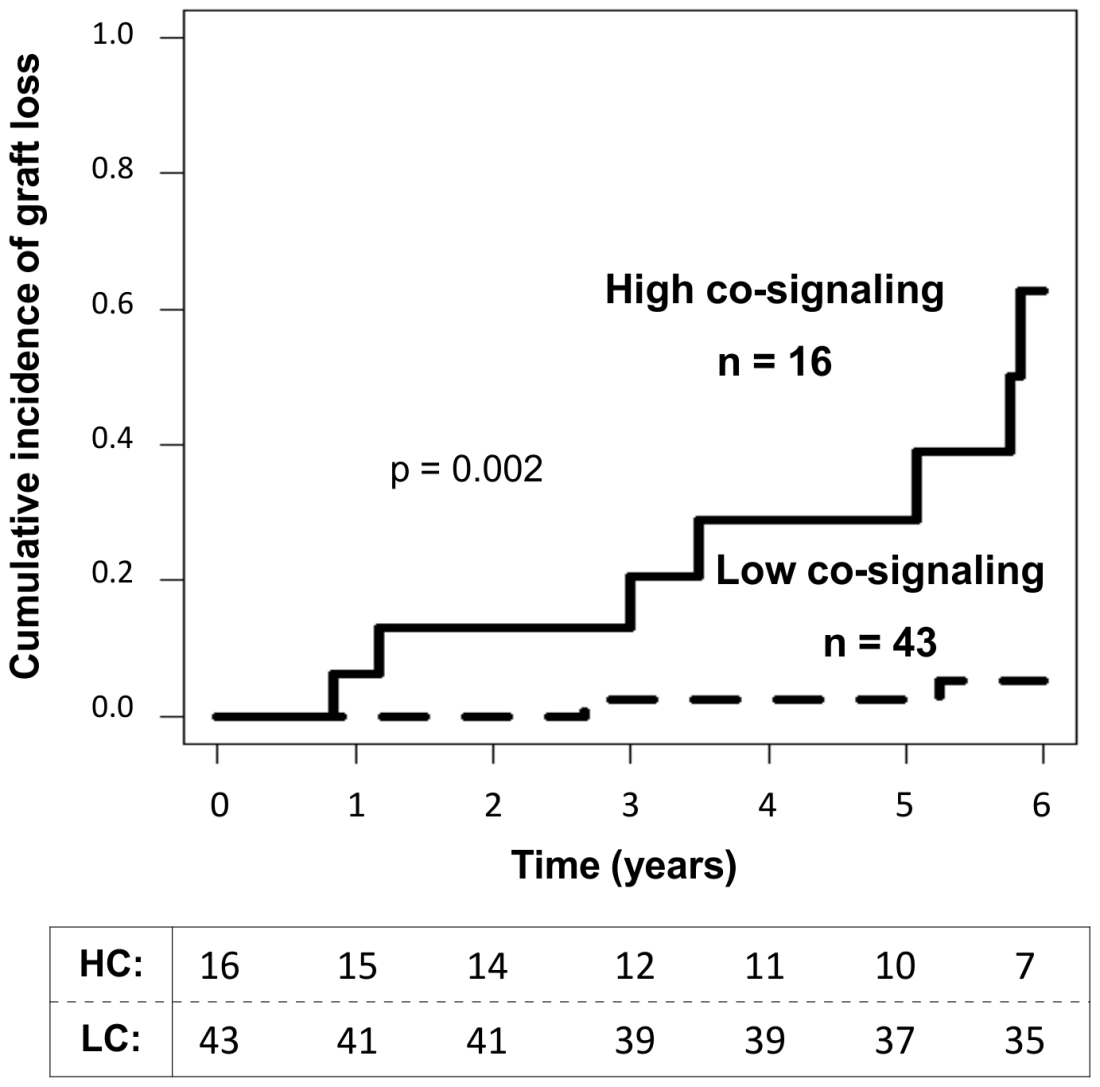
Long-term graft survival based on the determination of soluble co-signaling molecules. Patients with high levels of soluble co-signaling molecules determined at 3 months post-transplantation have a higher risk of graft failure than those with low levels (low co-signaling group) (p = 0.002). The Nelson-Aalen estimator was used to analyze the incidence of graft loss due to the existence of competing events (patients died with a functioning graft during the follow-up period). The number of patients analyzed each year, taking into account those with graft loss and those who died with a functioning graft are presented at the bottom.

We examined whether clustering of patients at 3 months post-transplantation enabled long-term graft outcome to be predicted. Using competing risk methodology, we estimated that the cumulative incidence of graft loss was significantly higher in the high co-signaling group than in patients with low co-signaling (HR 11.88, 95% confidence interval [CI], 2.458–57.402, p = 0.002) (**Fig. 3**). Patients with high values of the co-stimulatory and/or co-inhibitory components (>1.5) had a greater risk of graft failure at 6 years post-transplantation than patients with low levels (≤1.5) of these components (62.55% *versus* 5.14%, p<0.001). Data were adjusted by age of donor and recipient, sex, dialysis type, previous transplant and immunosuppressive therapy, indicating that the risk of graft failure in these patients was independent of these demographic and clinical characteristics.

**Figure 4:**
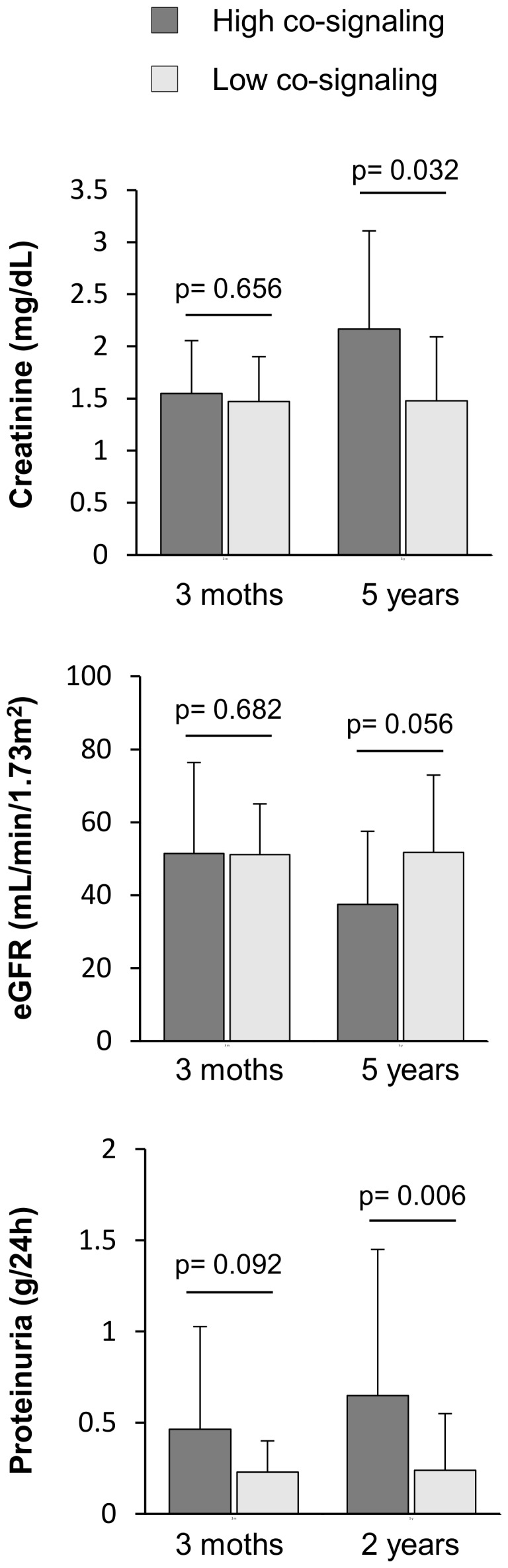
Changes of the creatinine, estimated glomerular filtration rate (eGFR) and proteinuria levels over time in kidney-transplanted patients grouped by the levels of soluble co-signaling molecules. Patients were clustered according to the levels of soluble co-signaling molecules quantified at 3 months post-transplantation in high co-signaling and low co-signaling groups. The levels of creatinine, eGFR and proteinuria did not differ between these two groups at time the soluble molecules were quantified. At 5 years post-transplantation, patients in the high co-signaling group showed an increase in creatinine levels and a decrease in the eGFR, whilst the characteristics of the patients in the low co-signaling group were unchanged. Changes in the proteinuria levels between both groups were observed by the second year post-transplantation.

Additionally, we analyzed long-term graft evolution in patients with a functioning graft using clinical parameters commonly used in clinical practice and based on non-invasive methods. For this, we considered the creatinine and proteinuria values and eGFR at 6 months and annually thereafter until the fifth year post-transplantation. These parameters were based on at least two successive measurements each year in order to avoid specific variations. We observed a slight and progressive increase in serum creatinine levels over time in the high co-signaling group, but unchanged levels in the low co-signaling group (**Table 3**). Consequently, patients in the high co-signaling group showed an sustained decrease in the eGFR (from 51.47±24.71 at 3 months to 37.53±20.00 mL/min/1.73 m^2^ 5 years after transplantation) whilst these values were stable in patients with low levels of co-signaling molecules determined at 3 months post-transplantation. However, differences in the proteinuria levels between the two groups were observed early on. At 1 year post-transplantation, a statistically significant difference was found between patients with high and low co-signaling molecules levels (0.52±0.60 *versus* 0.25±0.27 g/24 h), although in both cases the values were less than 0.5 g/24 h, which is considered clinically significant. This difference was more evident in later years, whereby patients in the high co-signaling group had higher proteinuria levels than patients with low co-signaling molecules. Thus, clustering of the patients based on the presence of co-signaling molecules at 3 months post-transplantation was independent of creatinine, eGFR and proteinuria values, but predicted the changes of these common markers of graft failure over time (**Fig. 4**).

**Table 3:** Creatinine, estimated glomerular filtration rate and proteinuria levels of both clusters of patients over time.

	High co-signaling			Low co-signaling				
Time (Post-tx)	Samples (n)	Mean ± SD	Median	Samples (n)	Mean ± SD		Median	p
**Creatinine levels (mg/dL):**								
3 months	16	1.55±0.51	1.45	43	1.47±0.43	1.40		0.656
6 months	16	1.59±0.49	1.55	43	1.41±0.41	1.36		0.183
1 year	15	1.77±0.87	1.40	41	1.42±0.36	1.40		0.281
2 years	14	1.58±0.45	1.41	41	1.41±0.41	1.38		0.179
3 years	12	1.65±0.48	1.53	39	1.36±0.44	1.35		0.076
4 years	11	1.64±0.46	1.68	39	1.42±0.48	1.34		0.125
5 years	10	2.16±0.94	2	37	1.48±0.61	1.32		**0.032**
**Glomerular filtration rate (eGFR) (mL/min/1.73 m^2^):**								
3 months	16	51.47±24.71	46.35	43	51.17±17.21	53.30		0.682
6 months	16	49.47±23.56	41.65	43	53.08±15.24	51.50		0.195
1 year	15	48.51±24.89	43.40	41	51.62±13.88	50.80		0.247
2 years	14	47.85±15.60	45.15	41	53.05±15.83	51		0.199
3 years	12	45.39±13.93	44.95	39	55.79±18.32	51.95		0.076
4 years	11	45.14±14.77	41.10	39	53.22±19.95	52.15		0.264
5 years	10	37.53±20.00	37.70	37	51.70±21.24	49.10		0.056
**Proteinuria levels (g/24 h):**								
3 months	16	0.46±0.56	0.25	43	0.23±0.17	0.20		0.092
6 months	16	0.45±0.47	0.22	43	0.24±0.19	0.17		0.061
1 year	15	0.52±0.60	0.28	41	0.25±0.27	0.14		**0.039**
2 years	14	0.65±0.80	0.39	41	0.24±0.31	0.16		**0.006**
3 years	12	0.89±1.07	0.43	39	0.34±0.40	0.18		**0.045**
4 years	11	1.04±1.53	0.39	39	0.40±0.70	0.21		0.191
5 years	10	1.38±1.58	0.89	37	0.42±0.62	0.21		0.108

Evolution of the kidney function in both clusters of patients determined by the levels of co-signaling molecules at 3 months post-transplantation. Medians were compared using Mann-Whitney U test and values of p <0.05 are shown in bold.

Similar results were observed when patients were classified simultaneously by clinical parameters, histological criteria and graft loss. Accordingly, we were able to define three groups: “Excellent long-term graft evolution”, defined as serum creatinine (sCr) ≤1.4 mg/dL, eGFR ≥60 mL/min/1.73 m^2^ and proteinuria ≤0.2 g/24 h, without donor-specific anti-HLA antibodies (HLA-DSA) and absence of AR or graft loss with a non-functioning graft; “Bad long-term graft evolution”, determined as serum creatinine (sCr) ≥2 mg/dL, eGFR ≤30 mL/min/1.73 m^2^ or proteinuria ≥1 g/24 h, and/or development of HLA-DSA, biopsy-proven AR or graft loss with a non-functioning graft. Patients that did not fall within either of these groups were classified as “Intermediate long-term graft evolution”. As we previously reported, creatinine, eGFR and proteinuria values were determined from at least two successive measurements each year. Combined analysis indicated that 68.75% (n = 11) of the high co-signaling patients showed bad graft evolution, whilst 44.18% (n = 19) of the patients in the low co-signaling group had an excellent evolution at 5 year post-transplantation (p<0.001) (**Table 4**). These patterns were still evident 4 years after transplantation (p = 0.029) (data not shown). Thus, the levels of these soluble molecules at 3 months after transplantation might be a biomarker enabling the early prediction of long-term graft acceptance.

**Table 4:** Effect of soluble co-signaling molecules determined at three months post-transplantation in the long-term (at 5 years post-transplantation) kidney graft evolution.

	GRAFT EVOLUTION		
	Bad	Intermediate	Excellent
**High co-signaling** n (%)	11 (68.75%)*	3 (18.75%)	2 (12.5%)
**Low co-signaling** n (%)	7 (16.27%)	17 (39.53%)	19 (44.18%)

The chi-square test was used to compare distributions among groups. * p <0.001.

## Discussion

The identification of biomarkers that indicate the immunological status of transplanted patients is an area of continuing interest in transplantation immunology. In this study, we found that high levels of soluble co-signaling molecules in the sera of kidney-transplanted patients determined at 3 months post-transplantation were significantly associated with long-term graft outcome. The quantification of these soluble molecules by a non-invasive method might reflect an activated immune system that negatively interferes with graft evolution, and will therefore be useful for the early identification of patients at high risk of graft loss before the damage becomes irreversible.

Co-stimulatory molecules are necessary for full T-cell activation, which, if not resolved, leads to a sustained and prolonged immune response that may trigger an inflammatory process involving the allograft. After initial T-cell activation, co-stimulatory molecules can be released in their soluble form, whereupon they function independently of membrane-binding molecules [27], [28]. The main purposes of this study were to determine whether soluble co-signaling molecules can be detected in transplanted patients and to understand how they are modulated during transplantation. We found that all patients had high levels of the soluble molecules related to T-cell activation (sCD30, sCD40 and sCD137) in the pre-transplantation samples, whilst only a few showed the presence of the soluble co-inhibitory molecules, PD-1 and PD-L1. It is known that cellular activation is exacerbated in dialysis patients, and this can contribute to the enhanced release of these molecules by activated T cells [29], [30]. Increased sCD40 levels in these patients enable competition with membrane-bound CD40 by CD40L engagement and compromise the humoral response to infections or vaccination [31], [32]. No association was found between the levels of these molecules previous to transplant and the type and time of dialysis, history of previous transplants or the cause of ESRD, suggesting that the aberrant expression of these soluble molecules in these patients might be due to the dialysis process by itself. In our study, only three patients were enrolled in a kidney transplant without previous dialysis treatment, so it is difficult to conclude that only continuous dialysis cycles increase the release of these soluble forms. Future studies analyzing the presence of these soluble molecules in ESRD patients with different eGFR levels would allow the initial presence of these molecules to be established so that their accumulation over time could be avoided. Most of these molecules have a high molecular weight (>30 kDa) and do not pass through the renal filter, so it is improbable that they will accumulate in the patient's serum due to the loss of the graft function. Indeed, a previous study showed that serum sCD30 values in dialysis patients were similar before, during or immediately after two dialysis cycles [33]. Patients with rheumatoid arthritis, where the kidney function is normal, showed a high presence of soluble PD-1 and PD-L1 associated with the persistent activation of self-reactive T cells [20]. Thus, we propose that the presence of high level of these soluble molecules is mainly due to an exacerbated immune activation and not by a loss of the renal filter function. Unexpectedly, we found lower sCD40L levels in pre-transplantation samples than in healthy controls. Platelets are the main resource of this molecule but conventional therapy with antiplatelet agents, statins or angiotensin-converting enzyme inhibitors might reduce its expression in these patients [34].

Two weeks after the kidney transplantation, the levels of these soluble molecules were significantly modified except for sPD-1 and sPD-L1, whose high levels were maintained in the same patients before and after transplantation. This suggests that the transplant itself or the direct effect of immunosuppressive treatment, which acts mainly on activated T-cells, could modify the expression of these molecules and thus their release into the blood. Nevertheless, we cannot rule out the possibility that some small molecules, such as sCD137 (16 kDa), could be filtered by the kidney after function recovery. Despite this, a group of transplanted patients maintain altered levels of these soluble molecules and never attained those of healthy controls, which may mean that a low but continuous stage of cellular activation is maintained in these patients, contributing to their progressive graft deterioration. In fact, we found that quantifying co-stimulatory (sCD30, sCD40, sCD137 and sCD40L) and co-inhibitory (sPD-1 and sPD-L1) molecules at 3 months post-transplantation identifies a group of patients with a worse 6-year graft outcome. Patients with high levels of soluble molecules (high co-signaling group) were associated with a 12-fold greater risk of subsequent graft loss. Moreover, all other patients with a functioning graft showed a continuous and progressive deterioration of kidney function over time, as it is reflected by the increased creatinine and proteinuria values and the gradual impairment of the eGFR. Although clustering of transplanted patients was observed even when soluble molecules were assayed 15 days post-transplantation, the best significant association with the clinical graft evolution was obtained with quantification 3 months after transplantation. This suggests that 3 months is the best time to predict graft outcome, whilst measurements at earlier times might exhibit variation arising from processes such as inflammation and immune activation by the transplantation event itself or by the period of adjustment required by the immunosuppressive therapy.

Thus, the quantification of these soluble molecules three months after transplantation reflects an immune landscape that could have consequences for long-term graft outcome. The persistence of soluble co-signaling molecules can modify the immune response post-transplantation before clinical manifestations can be detected. However, questions about the exact function of these soluble molecules in the alloimmune response remain unanswered.

The presence of high or low levels of soluble co-signaling molecules at three months post-transplantation was independent of the current serum creatinine levels or eGFR, which rules out the possibility that high levels of these molecules might reflect impaired graft function. Only a slight increase in the level of total proteins in the urine after 24 hours was observed in the high co-signaling group patients compared with those in the low co-signaling group. However, the difference was not statistically significant and, in any case, both values were less than the 0.5 g/24 h that is considered clinically significant. We observed that patients receiving hemodialysis had higher levels of soluble co-signaling molecules at 3 months post-transplantation. Some previous studies had found that continuous hemodialysis procedures produce important immunological changes leading to an aberrant state of T cell activation, a higher susceptibility of T cells to activation-induced cell death and a reduced proliferative T cell response [29], [35]. All these changes might be maintained after transplantation, modifying the profile of soluble co-signaling molecules post-transplantation [36]. Moreover, patients treated with tacrolimus as maintenance immunotherapy, in combination with steroids and an antiproliferative agent, were mainly clustered in the low co-signaling group. It is difficult to draw firm conclusions mainly because of the small number of patients and the variety of the immunosuppressive treatments, but the most immunosuppressed patients (thymoglobulin+ steroids+ tacrolimus + MMF) were more likely to feature in the low co-signaling group whilst patients with lower immunosuppression (Basiliximab + steroids + cyclosporine + mTOR or MMF) had higher levels of co-signaling molecules at 3 months post-transplantation. Thus, larger, multicenter studies are necessary to measure the effect of the immunosuppressive therapy in the presence of these soluble molecules and to establish whether the quantification of these molecules early post-transplantation might help guide decisions about adjusting the maintenance immunotherapy necessary for each patient.

In recent years, considerable importance has been attributed to the T-cell activation marker sCD30 [37], [38]. Several studies have shown an association between pre- and post-transplantation sCD30 levels and the development of AR and graft loss [39]–[44], although some questions remain unanswered [45], [46]. Süsal et al. [33] reported an interesting multicenter study in which a number of kidney-transplanted patients (9%) with high levels of sCD30 (≥40 U/ml) on day 30 showed worse graft survival. The high levels of this molecule were related to an activated alloimmune state. Consistent with this, we demonstrated that the combined effects of several co-signaling molecules allow the early detection of more patients at high risk (27.12% in our study) before irreversible graft damage ensued. Thus, further studies are needed to elucidate the role of additional co-signaling molecules in the graft evolution of kidney-transplanted patients.

Surprisingly, we observed that the co-inhibitory molecule PD-1 and its ligand (PD-L1) were present at higher levels in patients with a poor graft outcome. The soluble form of these molecules acts as an antagonist, blocking PD-1/PD-L1 engagement and increasing the proliferation and cytokine production of T cells [47]-[49]. To our knowledge, our study provides the first evidence of the presence of the sCD137 (4-IBB) molecule in transplanted patients. Engagement of CD137 with its ligand (CD137L) acts to amplify the existing co-stimulatory signals preferentially in CD8 T cells [50]–[52], although the exact role of sCD137 remains unknown.

We are aware that more, larger prospective longitudinal studies are required to validate our results. However, our findings show for the first time an aberrant expression of soluble co-signaling molecules in kidney-transplanted patients, which could condition the post-transplantation immune response. The algorithm developed here, based on the determination of six soluble molecules (sCD30, sCD40, sCD137, sCD40L, sPD-1 and sPD-L1) at 3 months post-transplantation, could be useful for the early identification of patients at high risk of poor graft outcome. Further studies will distinguish which of these soluble molecules play a key role in the immune response after transplantation.

## Supporting Information

Figure S1 **Clustering of patients by levels of co-signaling molecules assayed before transplantation and at 15 days post-transplantation.** Principal component analysis reduced the soluble molecule data to two principal components: the co-stimulatory (sCD30, sCD40, sCD137 and sCD40L) and the co-inhibitory (sPD-1 and sPD-L1). The dendrogram is derived from hierarchical clustering of all patients (n  =  59) and healthy controls (n = 25) based on the principal components from samples obtained before the transplantation (A) and 15 days post-transplantation (B). Each line represents a single kidney-transplanted patient (black) or healthy control (gray).
(TIF)

Table S1 **Quantification of soluble costimulatory molecules in serum from healthy donors and kidney-transplanted patients.**
(DOC)
