# Supplementary figures and images for: Soluble Co-Signaling Molecules Predict Long-Term Graft Outcome in Kidney-Transplanted Patients
Source: PLoS One. 2014 Dec 5;9(12):e113396. doi: 10.1371/journal.pone.0113396 (PMC4257538; doi:10.1371/journal.pone.0113396)

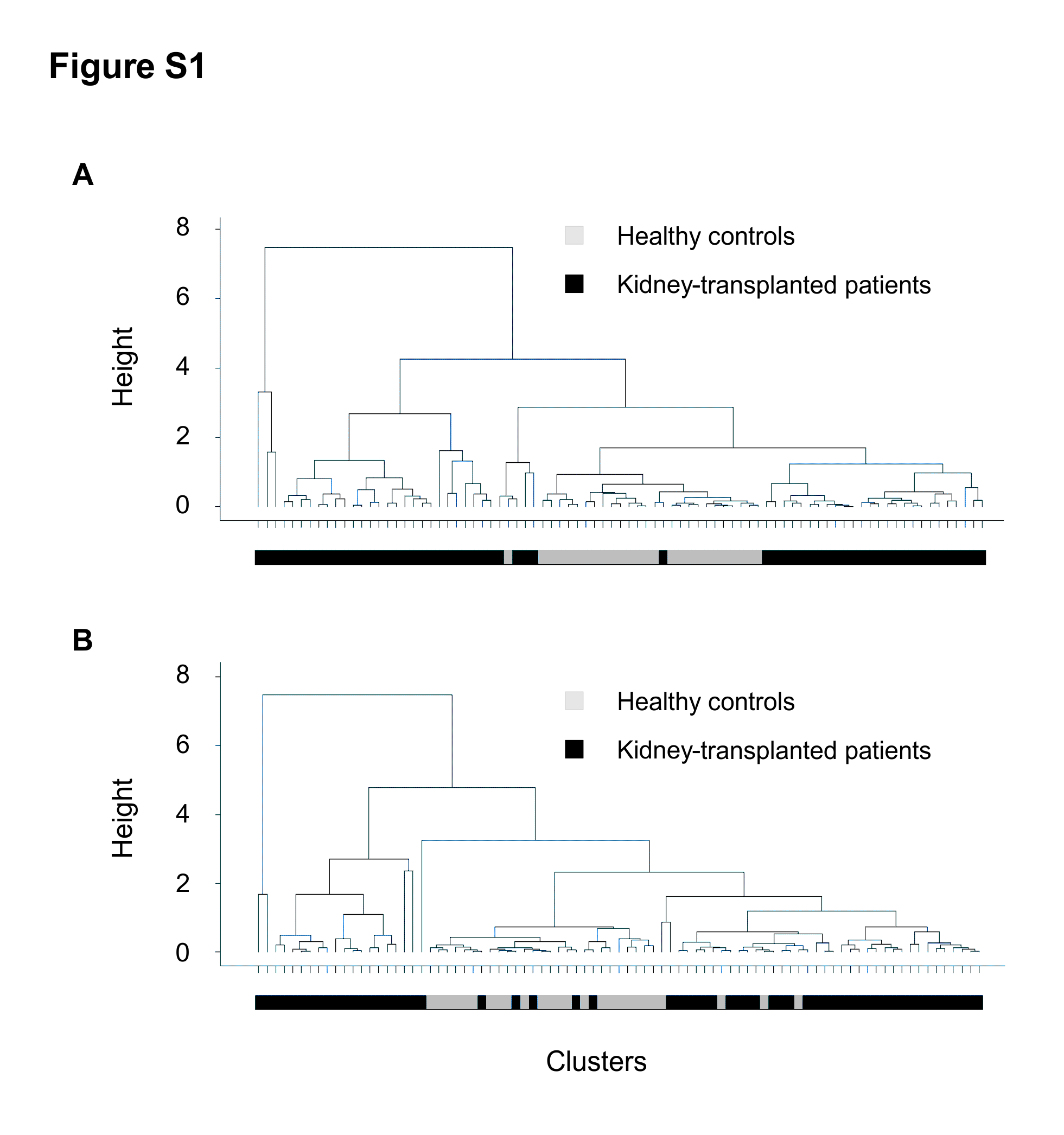

Supplement: Figure S1 — Clustering of patients by levels of co-signaling molecules assayed before transplantation and at 15 days post-transplantation. Principal component analysis reduced the soluble molecule data to two principal components: the co-stimulatory (sCD30, sCD40, sCD137 and sCD40L) and the co-inhibitory (sPD-1 and sPD-L1). The dendrogram is derived from hierarchical clustering of all patients (n = 59) and healthy controls (n = 25) based on the principal components from samples obtained before the transplantation (A) and 15 days post-transplantation (B). Each line represents a single kidney-transplanted patient (black) or healthy control (gray). (TIF) [file pone.0113396.s001.tif]
