# Supplementary material for: Soluble Co-Signaling Molecules Predict Long-Term Graft Outcome in Kidney-Transplanted Patients
Source: PLoS One. 2014 Dec 5;9(12):e113396. doi: 10.1371/journal.pone.0113396 (PMC4257538; doi:10.1371/journal.pone.0113396)
Supplement: Table S1 — Quantification of soluble costimulatory molecules in serum from healthy donors and kidney-transplanted patients. (DOC) [file pone.0113396.s002.doc]

**TABLE S1**. **Quantification of soluble costimulatory molecules in serum from healthy donors and kidney-transplanted patients.**

|  |  |  | **Post-transplantation** | | |
| --- | --- | --- | --- | --- | --- |
|  | **Healthy controls** | **Pre-transplantation** | **15 days** | **3 months** | **1 year** |
| **sCD30 (U/ml)** | |  |  |  |  |
| n | 25 | 59 | 59 | 59 | 58 |
| Mean ± SD | 17.86 ± 6.45 | 88.91 ± 39.11 | 36.43 ± 19.22 | 33.99 ± 23.2 | 36.6 ± 37.09 |
| Median | 17.86 | 83.36 | 33.77 | 28.46 | 26.27 |
| IQR | 13.58 - 23.14 | 59.5 - 107.68 | 21.30 - 45.83 | 18.75 - 42.12 | 18.30 - 38.93 |
| p (*****) |  | < 0.001 | < 0.001 | < 0.001 | < 0.001 |
| p (**†**) |  |  | < 0.001 | < 0.001 | < 0.001 |
| **sCD40 (pg/ml)** | |  |  |  |  |
| n | 25 | 59 | 59 | 57 | 57 |
| Mean ± SD | 24.68 ± 11.27 | 365.44 ± 344.73 | 69.78 ± 58.75 | 40.05 ± 25.18 | 62.54 ± 191 |
| Median | 23 | 250 | 58 | 39 | 25.2 |
| IQR | 19.00-28.50 | 143.35 - 510.71 | 34.14 - 85.50 | 20.40 - 51.43 | 8.40-50.00 |
| p (*) |  | < 0.001 | < 0.001 | 0.009 | 0.775 |
| p (**†**) |  |  | < 0.001 | < 0.001 | < 0.001 |
| **sCD137 (pg/ml)** | |  |  |  |  |
| n | 25 | 59 | 55 | 53 | 55 |
| Mean ± SD | 0.02 ± 0.09 | 2.84 ± 2.38 | 0.31± 0.55 | 0.28 ± 0.77 | 0.54 ± 0.9 |
| Median | 0 | 2.49 | 0 | 0 | 0 |
| IQR | 0 - 0 | 1.02 - 4.12 | 0 - 0.40 | 0 - 0.03 | 0 - 0.68 |
| p (*) |  | < 0.001 | 0.006 | 0.02 | 0.001 |
| p (**†**) |  |  | < 0.001 | < 0.001 | < 0.001 |
| **CD40L (ng/ml)** | |  |  |  |  |
| n | 25 | 57 | 58 | 55 | 53 |
| Mean ± SD | 8.41 ± 2.67 | 4.26 ± 3.01 | 6.19 ± 3.5 | 5.76 ± 3.42 | 6.9 ± 3.92 |
| Median | 9.08 | 3.86 | 5.61 | 5.21 | 6.77 |
| IQR | 6.93 - 10.23 | 1.79 - 5.95 | 3.93 - 7.23 | 3.73 - 7.6o | 3.86 - 9.07 |
| p (*) |  | < 0.001 | < 0.001 | < 0.001 | 0.018 |
| p (**†**) |  |  | 0.001 | 0.004 | 0.001 |
| **sPD-1 (ng/ml)** | |  |  |  |  |
| n | 25 | 59 | 55 | 47 | 47 |
| Mean ± SD | 1.41 ± 0.96 | 2.02 ± 2.89 | 4.12 ± 16.84 | 1.65 ± 2.78 | 1.69 ± 2.12 |
| Median | 1 | 0.83 | 0.72 | 0.63 | 0.81 |
| IQR | 0.73-1.95 | 0.43-1.96 | 0.39-1.34 | 0.34-1.5 | 0.4-1.95 |
| p (*) |  | 0.237 | 0.017 | 0.02 | 0.184 |
| p (**†**) |  |  | 0.353 | 0.771 | 0.671 |
| **sPD-L1 (ng/ml)** | |  |  |  |  |
| n | 25 | 57 | 58 | 55 | 47 |
| Mean ± SD | 1.48 ± 1.79 | 3.32 ± 8.42 | 3.12 ± 8.38 | 2.68 ± 6.73 | 2.17 ± 3.14 |
| Median | 0.85 | 0.9 | 0.95 | 0.9 | 1.13 |
| IQR | 0.52 - 1.82 | 0.52 - 2.84 | 0.43 - 2.08 | 0.45 - 1.98 | 0.30 - 2.72 |
| p (*) |  | 0.449 | 0.758 | 0.681 | 0.869 |
| p (**†**) |  |  | 0.262 | 0.181 | 0.318 |

Data are summarized as sample number (n), mean ± SD, median and interquartile range (IQR). Unpaired and paired Wilcoxon tests, significance indicated by p, were used to compare distributions of the soluble molecules between healthy controls and kidney-transplanted patients samples obtained before and after transplantation (*) and between patients samples obtained at different pre- and post-transplantation times (**†**), respectively.
